# Integrating population-level and cell-based signatures for drug repositioning

**DOI:** 10.1093/bioinformatics/btaf498

**Published:** 2025-09-10

**Authors:** Chunfeng He, Yue Xu, Yuan Zhou, Jiayao Fan, Chunxiao Cheng, Ran Meng, Lang Wu, Ruiyuan Pan, Ravi V Shah, Eric R Gamazon, Dan Zhou

**Affiliations:** The Second Affiliated Hospital and School of Public Health, Zhejiang University School of Medicine, 310058, HangZhou, China; The Key Laboratory of Intelligent Preventive Medicine of Zhejiang Province, Zhejiang University, 310058, HangZhou, China; The Second Affiliated Hospital and School of Public Health, Zhejiang University School of Medicine, 310058, HangZhou, China; The Key Laboratory of Intelligent Preventive Medicine of Zhejiang Province, Zhejiang University, 310058, HangZhou, China; Department of Biostatistics and Center for Quantitative Sciences, Vanderbilt University Medical Center, Nashville, TN 37232, United States; The Second Affiliated Hospital and School of Public Health, Zhejiang University School of Medicine, 310058, HangZhou, China; The Key Laboratory of Intelligent Preventive Medicine of Zhejiang Province, Zhejiang University, 310058, HangZhou, China; The Second Affiliated Hospital and School of Public Health, Zhejiang University School of Medicine, 310058, HangZhou, China; The Second Affiliated Hospital and School of Public Health, Zhejiang University School of Medicine, 310058, HangZhou, China; Cancer Epidemiology Division, Population Sciences in the Pacific Program, University of Hawaii Cancer Center, University of Hawaii at Manoa, Honolulu, HI 96813, United States; Key Laboratory of Mental Health of the Ministry of Education, Guangdong-Hong Kong-Macao Greater Bay Area Center for Brain Science and Brain-Inspired Intelligence, Southern Medical University, Guangzhou 510515, China; Guangdong-Hong Kong Joint Laboratory for Psychiatric Disorders, Guangdong Province Key Laboratory of Psychiatric Disorders, Southern Medical University, Guangzhou 510515, China; Guangdong Basic Research Center of Excellence for Integrated Traditional and Western Medicine for Qingzhi Diseases, Southern Medical University, Guangzhou 510515, China; Department of Neurobiology, School of Basic Medical Sciences, Southern Medical University, Guangzhou 510515, China; Division of Cardiology, Vanderbilt University Medical Center, Nashville, TN 37232, United States; Division of Genetic Medicine, Vanderbilt University Medical Center, Nashville, TN 37232, United States; Vanderbilt Diabetes Center, Vanderbilt University Medical Center, Nashville, TN 37232, United States; The Second Affiliated Hospital and School of Public Health, Zhejiang University School of Medicine, 310058, HangZhou, China; The Key Laboratory of Intelligent Preventive Medicine of Zhejiang Province, Zhejiang University, 310058, HangZhou, China

## Abstract

**Motivation:**

Drug repositioning presents a streamlined and cost-efficient way to expand the range of therapeutic possibilities. Drugs with human genetic evidence are more likely to advance successfully through clinical trials toward Food and Drug Administration approval. Single gene-based drug repositioning methods have been implemented, but approaches leveraging a broad spectrum of molecular signatures remain underexplored.

**Results:**

We propose a framework called “Transcriptome-informed Reversal Distance” (TReD) that embeds the disease signatures and drug response profiles into a high-dimensional normed space to quantify the reversal potential of candidate drugs in a disease-related cell-based screening. We applied TReD to COVID-19, type 2 diabetes, and Alzheimer’s disease (AD), identifying 36, 16, and 11 candidate drugs, respectively. Among these, literature supports 69% (25/36), 31% (5/16), and 64% (7/11) of the drugs, with clinical trials conducted for seven COVID-19 candidates and three AD candidates. In summary, we propose a comprehensive genetics-anchored framework integrating population-level signatures and cell-based screening that has the potential to accelerate the search for new therapeutic strategies.

**Availability and implementation:**

Source code and datasets considered in this study are available at Github (https://github.com/zdangm/TReD). An archived snapshot is deposited at Zenodo (https://doi.org/10.5281/zenodo.16791909).

## 1 Introduction

Conventional drug development is expensive in cost and time, spanning over a decade with substantial financial investment to go from target identification to therapeutics (Wouters *et al.* 2020). Drug repositioning represents a cost-effective strategy to extend the utility of existing drugs, leveraging previous studies on safety in preclinical and clinical studies to accelerate drug application.

Genetic evidence substantially improves the success rate of drug repositioning. Gene–disease associations may play a significant role in repositioning, since empirical data suggest that drug targets with genetic support are 2.6 times more likely to be successful (Minikel *et al.* 2024). Genome-wide, transcriptome-wide, and proteome-wide association studies (GWAS, TWAS) are powerful genetic tools to predict drug repurposing candidates: e.g. the transcriptome-wide significant association between *TYK2* and critical illness in COVID-19 (Pairo-Castineira *et al.* 2021) facilitated the drug repurposing of baricitinib, which has demonstrated therapeutic benefit in subsequent clinical trials (Rubin 2022). However, drug repositioning focused on a single gene target may not be sufficient to elicit a meaningful therapeutic response, as complex diseases involve intricate networks of pathways and interactions (Pushpakom *et al.* 2019).

Multitarget approaches are needed to tackle the complexity of disease mechanisms. The growing evidence for polypharmacology calls for the adoption of experimental–computational multitarget approaches (Paolini *et al.* 2006). Among genomics-based multitarget methods, signature matching and pathway or network mapping are commonly used (Pushpakom *et al.* 2019). In signature matching, a drug’s transcriptomic “signature” is compared with that of a disease to identify potential therapeutic candidates; for a given disease signature comprising genes with altered up/downregulation, a drug that reverses the disease-associated gene expression changes may hold therapeutic potential.

Current transcriptomic signature matching methods face limitations in causal inference. Most existing approaches rely on differential gene expression (DGE) to generate a list of disease-associated genes. However, the transcriptome perturbations identified by DGE are challenging to interpret as molecular consequences or causes of disease, with one recent study claiming a higher likelihood of implicated genes being of the former class (Porcu *et al.* 2021). Genetically anchored TWAS minimizes the problem of reverse causality. TWAS estimates genetically regulated expression using fixed genetic variants, such as expression Quantitative Trait Loci (eQTLs), and correlate these with phenotypic traits to identify disease-associated genes (Gamazon *et al.* 2015). By leveraging genetically determined rather than observed expression, TWAS reduces confounding from reverse causation, thereby facilitating the inference of potential causal relationships between gene expression and disease. Although DGE analysis cannot establish causal relationships, targeting downstream molecular effects of disease remains a valid therapeutic strategy. Integrating TWAS and DGE can enhance the precision of drug candidate screening.

Existing transcriptomic signature matching methods also inadequately capture multitarget alignment and underuse key resources. Methodologically, previous approaches [e.g. a widely adopted nonparametric pattern-matching method based on the Kolmogorov–Smirnov (KS) statistics (Lamb *et al.* 2006)] do not fully consider the pattern of alignment between the (transcript)omic features of drug induction and disease susceptibility, including, explicitly, genetically determined risk, within a multitarget or polygenic framework. Wu *et al.* (2022) applied PrediXcan models to find drug repurposing candidates using the Integrative Library of Integrated Network-based Cellular Signatures (iLINCS) platform. The latest release (CMAP LINCS 2020) of this platform contains over 3 million gene expression profiles with >80 000 perturbations and >200 cell lines. The resource is roughly a three-fold expansion relative to the previous version (2017) (Subramanian *et al.* 2017), and has yet to be fully utilized in drug repositioning efforts.

To address these gaps, here we developed Transcriptome-informed Reversal Distance (TReD), which fully leverages the expression changes of each gene within the transcriptomic signatures of both diseases and drugs. We integrate cell-based drug response profiles (CMAP LINCS 2020) and population-level disease signatures (TWAS and DGE) to identify drug repurposing candidates. For illustration, we applied the framework to search for drug repurposing candidates for COVID-19, type 2 diabetes (T2D), and Alzheimer’s disease (AD).

## 2 Materials and methods

### 2.1 Study overview

**Figure 1.**
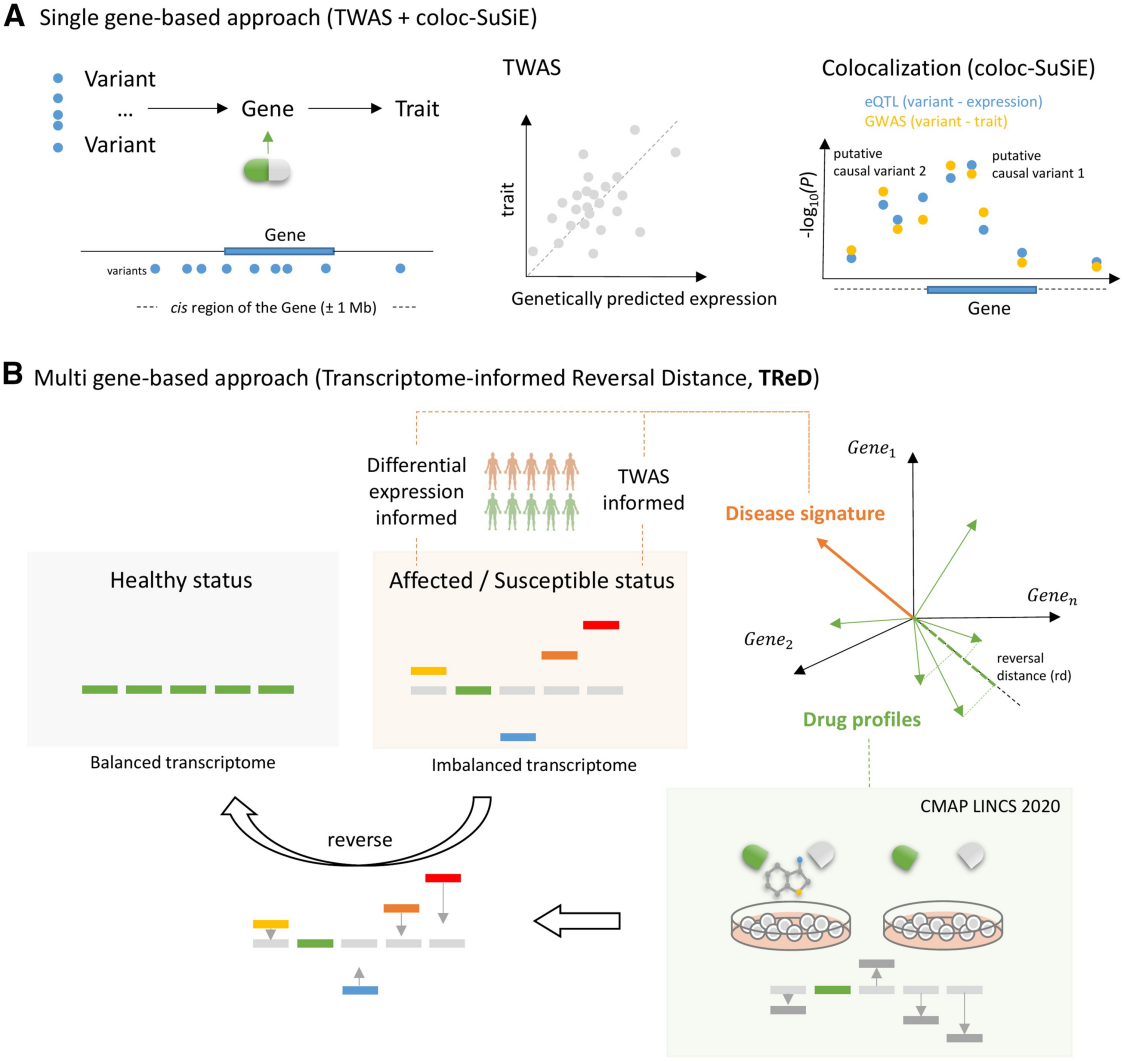
The framework overview. (A) Single gene-based approach integrates TWAS (e.g. PrediXcan) and colocalization analysis (e.g. coloc-SuSiE). (B) Multigene-based approach TReD (Transcriptome-informed Reversal Distance) utilizes DGE- and TWAS-derived population-level gene expression profiles as disease signatures, reflecting affection status and germline susceptibility, respectively. By embedding the population-level disease signatures and the cell-based drug response profiles in the same high-dimensional space, the reversal distance (*rd*) is estimated for each drug. A permutation test is used to estimate the statistical significance of the reversal distance.

Genetics-informed single-gene matching and omics profile-based signature matching are two promising approaches used in drug repurposing, with diverse types of omics data providing a valuable opportunity. In this study, we integrated these two approaches, leveraging transcriptome data to identify potential drug repurposing candidates (Fig. 1). A single gene-based approach that combines TWAS and colocalization analysis (coloc-SuSiE) detects genes associated with disease (Fig. 1A, Fig. 1, available as supplementary data at *Bioinformatics* online). Subsequently, drugs targeting these genes are prioritized as candidate therapeutics. For the multi-gene-based approach, we developed TReD, integrating TWAS- and DGE-derived disease signatures and cell-based drug response profiles (CMAP LINCS 2020) (Fig. 1B, Fig. 1, available as supplementary data at *Bioinformatics* online). In this study, we focused primarily on the drug response profile data arising from small-molecule perturbations in immune-related cells and blood glucose metabolism-related tissues for the specific TReD applications to COVID-19 and T2D, respectively. TReD is based on transcriptome signature matching, which relies on the signature reversion principle (Wagner *et al.* 2015). In contrast to previous transcriptome signature pattern-matching studies, we proposed a “reversal distance” which quantifies the drug’s potential in reversing the disease-associated gene expression, using a metric that is scale-invariant and directionality-focused. To conduct hypothesis testing and evaluate the statistical significance, we employed a permutation approach.

### 2.2 GWAS for COVID-19 and T2D

The GWAS summary statistics for COVID-19 severity were downloaded from COVID-19 Host Genetics Initiative (https://www.covid19hg.org/results/r7/), the latest version, release 7 of the hospitalized versus population data. The hospitalized phenotype refers to hospitalization with a laboratory confirmation of SARS-CoV2 infection. This dataset, predominantly of European ancestries, includes 44 989 cases and 2 356 386 controls. To ensure ancestry matching, we used European samples from the 1000 Genomes Project as the LD reference panel. The summary statistics of GWAS of T2D were obtained from Xue *et al.*’s study. This dataset contains 62 892 T2D cases and 596 424 controls of European ancestry (Xue *et al.* 2018) (http://cnsgenomics.com/data/t2d/). Additional descriptions of the included studies can be found in Table 7, available as supplementary data at *Bioinformatics* online. These GWAS summary statistics for COVID-19 and T2D were subsequently used as the input data for TWAS and colocalization analyses, as described in the following section.

### 2.3 TWAS and colocalization for COVID-19 and T2D

Building upon the GWAS summary statistics described above, we performed TWAS and colocalization analyses to prioritize genes with potential causal roles in COVID-19 and T2D. To identify disease-associated genes, we applied TWAS using the best prediction model selected from PrediXcan (Gamazon *et al.* 2015), UTMOST (Hu *et al.* 2019), and JTI (Zhou *et al.* 2020) for each gene. Model performance was evaluated by five-fold cross-validation. Models with cross-validated $R^{2}\geq 0.01$ and P≤.05 were excluded. Among the remaining models, the one with the highest $R^{2}$ was selected for downstream analysis, as a higher $R^{2}$ indicates better prediction accuracy of gene expression and is expected to yield greater statistical power in subsequent association testing. We used the residuals of the normalized gene expression after regressing out relevant covariates. Single-nucleotide polymorphisms (SNPs) in the *cis* region (i.e. 1 Mb upstream and downstream) of the gene were considered candidate features for the gene’s prediction models. Applying the prediction model to GWAS dataset, we would estimate the genetically predicted expression level for each individual. The significance of the effect of the predicted expression of a gene on a trait was determined by linear regression. In practice, summary statistics-based association test was performed. Summary statistics-based association test utilizes the weights from the prediction model of the gene under consideration, the covariances of the SNPs included in the prediction model for the gene, and the GWAS effect size coefficient for each SNP. Notably, the summary statistics-based approach results in nearly perfect concordance with the individual-level-based approach (Barbeira *et al.* 2018).

SARS-CoV-2 directly attacks pulmonary alveolar cells and causes lung injury. Pulmonary infections leading to COVID-19 induce adaptive immune responses, while effector immune cells are generated and recruited in the lymphocytes, spleen, and blood. Therefore, blood, lung, lymphocytes, and spleen were considered as relevant tissues for COVID-19. Visceral adipose, liver, pancreas, and skeletal muscle were considered relevant tissues for T2D. For TWAS results that passed Bonferroni multiple testing correction, we performed colocalization to provide additional support under different sets of assumptions. To perform colocalization analysis in the presence of multiple potentially causal variants, we applied coloc (Giambartolomei *et al.* 2014) to the GWAS hits decomposed by SuSiE (Wallace 2021) in each GWAS locus, which spans the SNPs located within 200 kb (in each direction) of the lead SNP. Notably, *coloc-SuSiE* is more generalizable since it relaxes the “one causal variant” assumption of regular *coloc*. We used European individuals in the 1000 Genomes Project as the LD panel. If SuSiE failed to converge after 1000 iterations for either eQTL or GWAS summary statistics, we instead used the regular *coloc*. eQTL and GWAS signals were determined to “colocalize” if the maximum posterior probability of colocalization (i.e. PP.H4) was greater than or equal to 0.85 using either *coloc* or *coloc-SuSiE*. TWAS significant genes showing positive colocalization evidence were then used to search for drugs targeting the genes, via DrugBank, Therapeutic Target Database (TTD), Drug Gene Interaction Database (DGIdb). DrugBank is a comprehensive and up-to-date resource for searching over 500 000 drugs and drug products, their targets, pathways, indications, and pharmacology. TTD is a comprehensive database of therapeutic targets, drugs, biomarkers and scaffolds for various diseases and International Classification of Diseases (ICD) identifiers. DGIdb is an online resource on drug–gene interactions and the druggable genome.

### 2.4 DGE for COVID-19 and T2D

In COVID-19, the DGE data—consisting of independent gene expression signatures labeled “ALV,” “EXP,” “BALF”—were derived from the study of Le *et al.* (2021). The ALV signature was generated from RNA-seq data in human adenocarcinomic alveolar basal epithelial cells (GEO Dataset: GSE147507; *n* = 67) (Table 8, available as supplementary data at *Bioinformatics* online). After Benjamini–Hochberg adjustment, the study identified 120 differentially expressed genes—100 upregulated and 20 downregulated. The EXP signature was obtained by RNA-seq on organoid samples (Table 9, available as supplementary data at *Bioinformatics* online) (GSE149312; *n* = 22). After Benjamini–Hochberg adjustment and applying a fold-change cutoff (|log2 FC|> 2), 125 genes remained significant. The BALF signature was estimated by RNA-seq analysis of the bronchoalveolar lavage fluid samples from two COVID-19 patients and three controls. After DESeq2 processing, 1349 genes were used to define the BALF signature (Table 10, available as supplementary data at *Bioinformatics* online).

Three DGE datasets (labeled as “islets,” “myoblasts,” and “myotubes”) were included in the T2D analysis. The islet signature was generated from microarray data on pancreatic islets derived from T2D cases and normal glucose-tolerant controls (Gunton *et al*. 2005), consisting of 370 differentially expressed genes at *P* < .01 (Table 11, available as supplementary data at *Bioinformatics* online). The other two signatures were obtained from the Davegårdh *et al.*’s study (Davegårdh *et al.* 2021). Based on a false discovery rate <5%, the study identified 577 differentially expressed genes in myoblasts and 42 differentially expressed genes in myotubes (Tables 12 and 13, available as supplementary data at *Bioinformatics* online).

### 2.5 The CMAP LINCS 2020 dataset

The Library of Integrated Network-Based Cellular Signatures (LINCS) is a National Institutes of Health Common Fund program. Its primary goal is to generate a comprehensive atlas of perturbation-response signatures. The CMAP LINCS 2020 data contain drug response profiles (defined by drug-induced gene expression profiles in response to perturbations). Here, we mainly focused on the transcriptomic response to small-molecule (drugs/compounds) perturbations in immune-related cells and blood glucose metabolism-related tissues for the application of the TReD to COVID-19 and T2D, respectively. We downloaded the Level 5 beta data consisting of drug response profiles arising from small-molecule perturbations from CMAP LINCS 2020 (https://clue.io/data/CMap2020#LINCS2020).

### 2.6 Candidate drug prioritization

To quantify the extent of reversal on disease of a specific drug, we used the cosine similarity metric. For a given disease, we first represent the set of disease signature as a vector in a high-dimensional space (whose dimension n is the total number of genes) with each nonzero element of the vector given by a disease-associated gene. The disease signature can be derived from either TWAS or DGE. The vector for each candidate drug is embedded in the same n-dimensional space. For each disease signature vector and drug response profile pair, we compute the reversal distance. Reversal distance measures a drug’s ability to counteract disease-related gene expression. A positive value suggests potential preventive or therapeutic effects, while a negative value indicates the drug may worsen the disease. A higher positive reversal distance suggests a stronger counteracting effect. Let $\vec{G\;}$ denotes a disease signature vector, $\vec{D\;}$ a drug profile vector, θ the angle between $\vec{G\;}$ and $\vec{D\;}$, and $|\left|\vec{a}\right||=\sqrt{\vec{a}\;.\vec{a}}$ the Euclidean norm of the vector $\vec{a}$. The cosine similarity ω is obtained by


(1) $$\omega :\;=\cos\theta =\frac{\vec{G\;}\;.\;\vec{D\;}}{\left|\left|\vec{G\;}\right|||\left|\vec{D\;}\right|\right|}\;$$

Then, the reversal distance rd, which is the reversal projection distance of $\vec{G\;}$ on $\vec{D\;}$, is calculated to quantify the potential effect of the drug in reversing the disease signatures:


(2) $$rd=|\left|\vec{G\;}\right||\cos\left(\pi -\theta \right)\;$$

Here, the change in ω in response to a perturbation in the ith element of the drug response profile $\vec{D}$, $d_{i}$, or the ith element of the gene signature $\vec{G}$, $g_{i}$, is given by


(3) $$\frac{\partial \omega }{\partial d_{i}}=\frac{g_{i}}{|\left|\vec{G\;}\right||\left|\left|\vec{D\;}\right|\right|}-\frac{d_{i}(\vec{G\;}\;.\;\vec{D\;})}{|\left|\vec{G\;}\right||{\left|\left|\vec{D\;}\right|\right|}^{3}}\;$$

(4) $$\frac{\partial \omega }{\partial g_{i}}=\frac{d_{i}}{|\left|\vec{G\;}\right||\left|\left|\vec{D\;}\right|\right|}-\frac{g_{i}(\vec{G\;}\;.\;\vec{D\;})}{|\left|\vec{D\;}\right||{\left|\left|\vec{G\;}\right|\right|}^{3}}\;$$

Note that the cosine similarity ω  (1) always lies in [-1,1], with −1 indicating exactly opposite orientation of the disease signature and the drug response profile and +1 indicating parallel orientation. Each quantity measures the sensitivity of the metric. In Equation (2), θ, and thus the reversal distance rd, is a function of $\vec{G\;}$ and $\vec{D\;}$. When we wish to emphasize this dependence, we will write rd as $rd(\vec{G\;},\vec{D\;})$, which defines a distance function. The distance function measures the similarity between abstract objects, and its properties enable this similarity metric to be used in comparisons. We obtain Equations (5) and (6) by rewriting Equation (2), it can be evidenced as follows:


(5) $$\cos(\pi -\theta )=\frac{rd(\vec{G\;},\vec{D\;})}{\left|\left|\vec{G\;}\right|\right|}\;$$

(6) $$\pi -\theta =\arccos(\frac{rd(\vec{G\;},\vec{D\;})}{\left|\left|\vec{G\;}\right|\right|})\;$$

Introducing a 2/π to normalize the angle π-θ to a distance from 0 to 2, we obtain Equation (7):


(7) $$d(\vec{G\;},\vec{D\;}):=\frac{2\arccos(rd(\vec{G\;},\vec{D\;})/|\left|\vec{G\;}\right||)}{\pi }\;$$

In Equation (7), $rd(\vec{G\;},\vec{D\;})/|\left|\vec{G\;}\right||\in [-1,\;1],\;$thus $\arccos(rd(\vec{G\;},\vec{D\;})/|\left|\vec{G\;}\right||)\in [0,\pi ]$, and$\;d(\vec{G\;},\vec{D\;})$ satisfies the conditions for a distance function, namely, non-negativity: $d(\vec{a},\vec{b})\geq 0$; symmetry: $d(\vec{a},\vec{b})=d(\vec{b},\vec{a})$; the triangle inequality; and the coincidence axiom: $d(\vec{a},\vec{b})=0$ if and only if $\vec{a}=\vec{b}$. Despite the symmetry of the distance function, there is clearly an underlying *asymmetry*, as the disease signature vector $\vec{G\;}$ determines which genes are the “relevant” elements of the drug response profile vector $\vec{D\;}$.

Of interest to us is the sampling distribution of the cosine similarity ω. Consider the function $h(s,\;t,\;u)=s/\sqrt{tu}$. Note that ω is equal to $h(\vec{G}\;.\;\vec{D},\;\vec{G}\;.\;\vec{G},\;\vec{D}\;.\;\vec{D})$. Furthermore, $\vec{G}\;.\;\vec{G}$ and $\vec{D}\;.\;\vec{D}$ are $\chi^{2}(n)$ distributed. We leverage the so-called Delta Method. Using the first two terms of the Taylor series expansion and setting Γ=(s, t, u) with expected value γ=E[Γ], we obtain approximate expressions for the mean and variance of h(Γ):


$$E[h(\Gamma )]\approx h(\gamma )+{\nabla h(\gamma )}^{T}\;.\;E[\Gamma -\gamma ]$$

$$\mathrm{Var}(h(\Gamma ))\approx {\nabla h(\gamma )}^{T}.\;\mathrm{Cov}(\Gamma )\;.\;\nabla h(\gamma ),$$

where ∇h is the gradient.

In this framework, drug repurposing is a search problem: the vector $\vec{G\;}$ is evaluated against all possible $\vec{D\;}$, which are prioritized using rd. A larger positive rd indicates a stronger reversal effect. Assuming K total number of drugs tested and the reversal distance for the ith drug, $rd(\vec{G\;},\vec{D_{i}})$, we get a probability distribution consisting of the K probabilities $p_{i}$ conditional on the disease signature information:


$$p_{i}=P(\vec{D_{i}}\;|\vec{G\;})=\frac{\exp(rd(\vec{G\;},\vec{D_{i}}))}{\sum_{j=1}^{K}\exp(rd(\vec{G\;},\vec{D_{j}}))}$$

by applying the softmax function. One drug repurposing approach is to identify the drug that maximizes the reversal distance:


$$D^{*}\;=\underset{\vec{D}}{\mathrm{argmax}}\left(\ln\left(\left|\left|\vec{G\;}\right|\right|\right)+\ln(\cos\left(\pi -\theta (\vec{G\;},\;\vec{D\;})\right))\right)$$

Here, we instead prioritized a potentially larger set of drugs, i.e. the top candidates that meet a significance threshold.

We converted the gene–disease association signals into rank ratios, which, for each gene, is defined as the rank of the gene divided by the total number of genes. Similarly, each element of the drug response profile was rank-transformed. Since all rank ratios range between 0 and 1, the cosine similarity calculated using these values also falls between 0 and 1. To more intuitively interpret cosine similarity in terms of synergistic versus antagonistic effects between two vectors, we subtracted 0.5 from each rank ratio, shifting the range to −0.5 to 0.5. This transformation allows the resulting cosine similarity to span from −1 to 1.

For each drug response profile, the *P*-value of the reverse distance (*rd*) is estimated by a permutation test. To generate the null distribution, we randomly shuffle the labels 1000 times to create numerous permuted samples. These samples are generated under the assumption of the null hypothesis, meaning there is no effect or association. In practice, the rank-ratio of disease signature is shuffled randomly. A null reversal distance is calculated on the basis of the shuffled disease signature. Then, we calculate the real reversal distance and estimate the proportion of permuted reversal distance that are as extreme or more extreme than the reversal distance obtained from the real data. This proportion represents the *P*-value. For each of the disease signatures, a significant drug response profile is defined as showing nominal significance in the permutation test (*P *< .05) and large rd (over 2SDs from the mean across all the drug response profiles). In addition, we further adjust the experiment-wise permutation *P*-value (pooling different perturbations) across the tested drugs using Bonferroni correction. For each disease, drugs with at least one significant (*P*_adj_<.05) drug response profile were prioritized.

We also implemented a degree-preserving permutation approach. Let G=(V, E) be a graph, where V is the set of nodes (genes) and E is the set of edges (defined by a coexpression network in a given tissue). To generate a permutation which preserves the degree distribution of the network, we randomly select two edges (say, a↔b and c↔d) with nonoverlapping nodes, remove the edges between the nodes, and create new edges (a↔c and b↔d) by swapping the nodes until a certain proportion (80%) of the edges have been shuffled. The null reversal distance is computed for each such permutation.

### 2.7 Application of TReD to COVID-19 and T2D

Leveraging the TWAS/DGE results and the cell-based gene expression data from CMAP LINCS 2020, we implemented the TReD framework to prioritize drug repositioning candidates for COVID-19 and T2D. Immune-related and T2D-related cell lines (matching the T2D-related tissues) have been annotated by multiple resources including the CMAP LINCS 2020 and the Cancer Cell Line Encyclopedia [cancer cell lines which have been used to study COVID-19 and other diseases (Bakowski *et al.* 2021)]. Information on drugs and drug targets is curated in the bioinformatics and chemoinformatics resource DrugBank (https://go.drugbank.com/releases/latest). A total of 1112 drugs and 12 immune-related cell types were included for COVID-19, while 1980 drugs and 10 related cell types were included for T2D.

We leveraged the TWAS results of multiple tissues and three DGE profiles as disease signatures (as defined in the previous section). For perturbations with repeated LINCS experiments, consistent direction of *rd* is required, i.e. drug response profile with repeated experiments showing inconsistent direction of *rd* will be removed. Considering the multiple testing, we prioritize drugs which show at least one significant drug response profile (*P*_adj_ <.05) for each disease.

### 2.8 Further validation of the TReD method: application to AD

The GWAS summary statistics for AD were obtained from Bellenguez *et al.*’s (2022) study. This dataset contains 111 326 AD cases and 677 663 controls of European ancestry (GWAS Catalog accession no. GCST90027158). We performed TWAS analyses on eight AD-related brain tissues to identify candidate risk genes, including the amygdala, caudate, hippocampus, hypothalamus, nucleus accumbens, cortex (general), cortex BA9, and cortex BA24. Details of the TWAS methodology have been described previously.

We combined TWAS results of the eight tissues with drug response profiles of 236 compounds from the CAMP LINCS 2020 dataset. These profiles were generated in eight AD-relevant cell lines. We applied the data to the TReD pipeline to identify potential drugs for AD.

### 2.9 Comparison of TReD with the KS statistic-based pattern-matching approach

We also conducted a comparative analysis between TReD and the KS statistic-based pattern-matching method (Le *et al.* 2021) on the same datasets to evaluate their performance in terms of sensitivity and specificity. Disease-associated genes were ranked based on the magnitude of expression changes, and gene-rank values range from 0 to 1. KS test splits the disease signature into two lists containing only upregulated genes (gene rank <0.5) and downregulated genes (gene rank ≥ 0.5). KS quantified the enrichment of disease-upregulated genes at the top and downregulated genes at the bottom of the drug-induced gene-rank list using cumulative distribution differences, yielding *ks_up* and *ks_down*, respectively. The final connectivity score was defined as *ks_up*, *–ks_down*, or *ks_up—ks_down*, depending on the available gene sets. Positive scores indicate potential reversal of disease-associated expression, while negative scores suggest possible exacerbation.

## 3 Results

### 3.1 Single-target drug repositioning using TWAS and coloc-SuSiE

**Figure 2.**
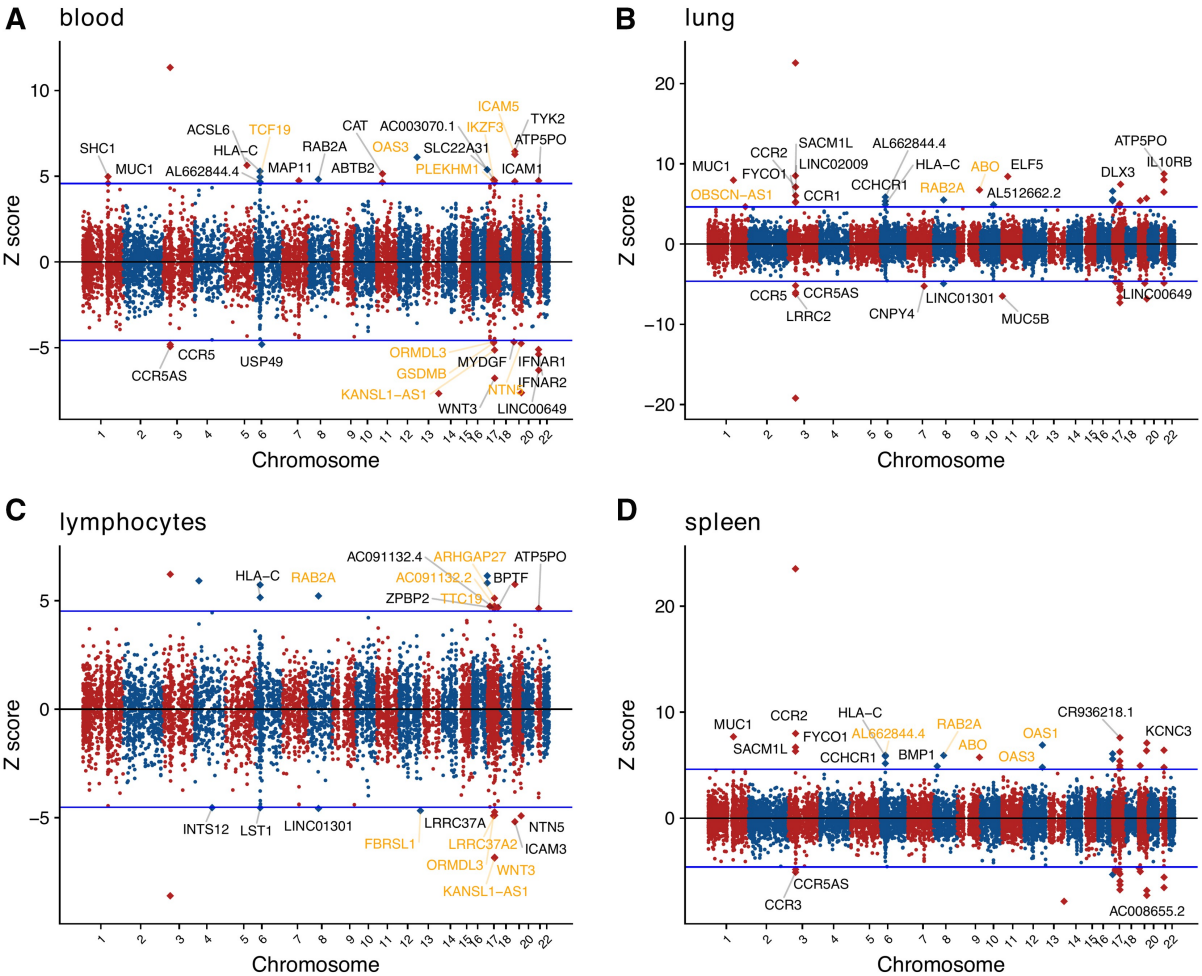
TWAS and colocalization analysis for COVID-19. Manhattan plots of TWAS results for COVID-19 severity in whole blood (A), lung (B), lymphocytes (C), and spleen (D), respectively. The *Z*-scores for TWAS are shown. *Z*-score >0 indicates a gene for which higher genetically determined expression is associated with COVID-19 severity. The labeled genes survived multiple testing corrections (over or below the blue lines) given the number of genes tested. Genes that further pass the significance threshold from the colocalization analysis are colored gold.

We performed TWAS to identify association between gene expression and a given phenotype. Models to predict gene expression were fit using a reference panel, subsequently applying gene expression models to GWAS of COVID-19 severity and T2D. We used data from Genotype-Tissue Expression v8, focusing on select tissues potentially relevant to COVID-19 severity [whole blood, lung, lymphocytes, and spleen samples (Vanderbeke *et al.* 2021)] or T2D (visceral adipose, liver, pancreas, skeletal muscle). Genes that survived Bonferroni correction for each tissue were considered significant (Fig. 2, Table 1, available as supplementary data at *Bioinformatics* online). For TWAS results significant after multiple testing, we performed coloc/coloc-SuSiE. Based on commonly used standards in the literature, eQTL and GWAS signals were determined to “colocalize” if maximum posterior probability of colocalization (i.e. PP.H4) was ≥0.85 for coloc or coloc-SuSiE. We also compared thresholds of 0.8 and 0.9, and found no significant difference in candidate genes identified across these thresholds. The results of coloc are in Table 2, available as supplementary data at *Bioinformatics* online and those of coloc-SuSiE are in Table 3, available as supplementary data at *Bioinformatics* online. We further examined which prioritized disease-related genes might be drug targets. Drug target genes were defined using public databases, including DrugBank (Wishart *et al.* 2018), the DGIdb (Freshour *et al.* 2021), and the TTD (Zhou *et al.* 2022).

For COVID-19 severity, we identified 34, 45, 27, and 43 significant genes in blood, lung, lymphocytes, and spleen, respectively in TWAS. Nine genes showed significance across four tissues. Notably, *tyrosine kinase 2* (*TYK2)* was significant in all tissues with a concordant positive effect, signifying that an increased expression of *TYK2* would be associated with an increased risk of severe COVID-19. TYK2 is an intracellular kinase belonging to the Janus kinase (JAK) family, which plays a pivotal role in the Janus Kinase–Signal Transducer and Activator of Transcription (JAK-STAT) pathway (Hu *et al.* 2021). High expression of *TYK2* and host-driven inflammatory lung injury are related to life-threatening COVID-19 (Pairo-Castineira *et al.* 2021). *RAB2A* also showed a positive effect in TWAS in the four tissues. *RAB2A* is required for SARS-CoV-2 replication (Li *et al.* 2020), and higher expression of *RAB2A* is associated with poorer COVID-19 outcome (Pairo-Castineira *et al.* 2023), consistent with directionality we observed.

In the four analyzed tissues, we identified a total of 28 unique genes post coloc/coloc-SuSiE (Fig. 2, available as supplementary data at *Bioinformatics* online). Among these targets, 50% (14/28) have been reported to be associated with COVID-19. *DPP9* (Wang *et al.* 2021a), *WNT3* (Wu *et al.* 2021), *ABO* (Ellinghaus *et al.* 2020), *NAPSA* (Krishnamoorthy *et al.* 2023), *GSDMB* (Chang *et al.* 2022), *RAB2A* (Pairo-Castineira *et al.* 2023), *OAS1* (Banday *et al.* 2022), *OAS3* (Pairo-Castineira *et al.* 2021), *LRRC37A2* (Zhu *et al.* 2023), *MAPT* (Cao *et al.* 2023), *FUT2* (Kousathanas *et al.* 2022), *IKZF3* (Yao *et al.* 2023), *ICAM5* (Kousathanas *et al.* 2022), *CCR9* (Ellinghaus *et al.* 2020). *CCR9* and *MAPT* are druggable genes: drugs such as docetaxel and astemizole, which target *MAPT*, has COVID-19-related support. Docetaxel can inhibit the active site of SARS-CoV-2 RdRP (Bagabir 2023). Astemizole (antihistamine) can interfere with SARS-COV-2 entry (Wang *et al.* 2021b).

**Figure 3.**
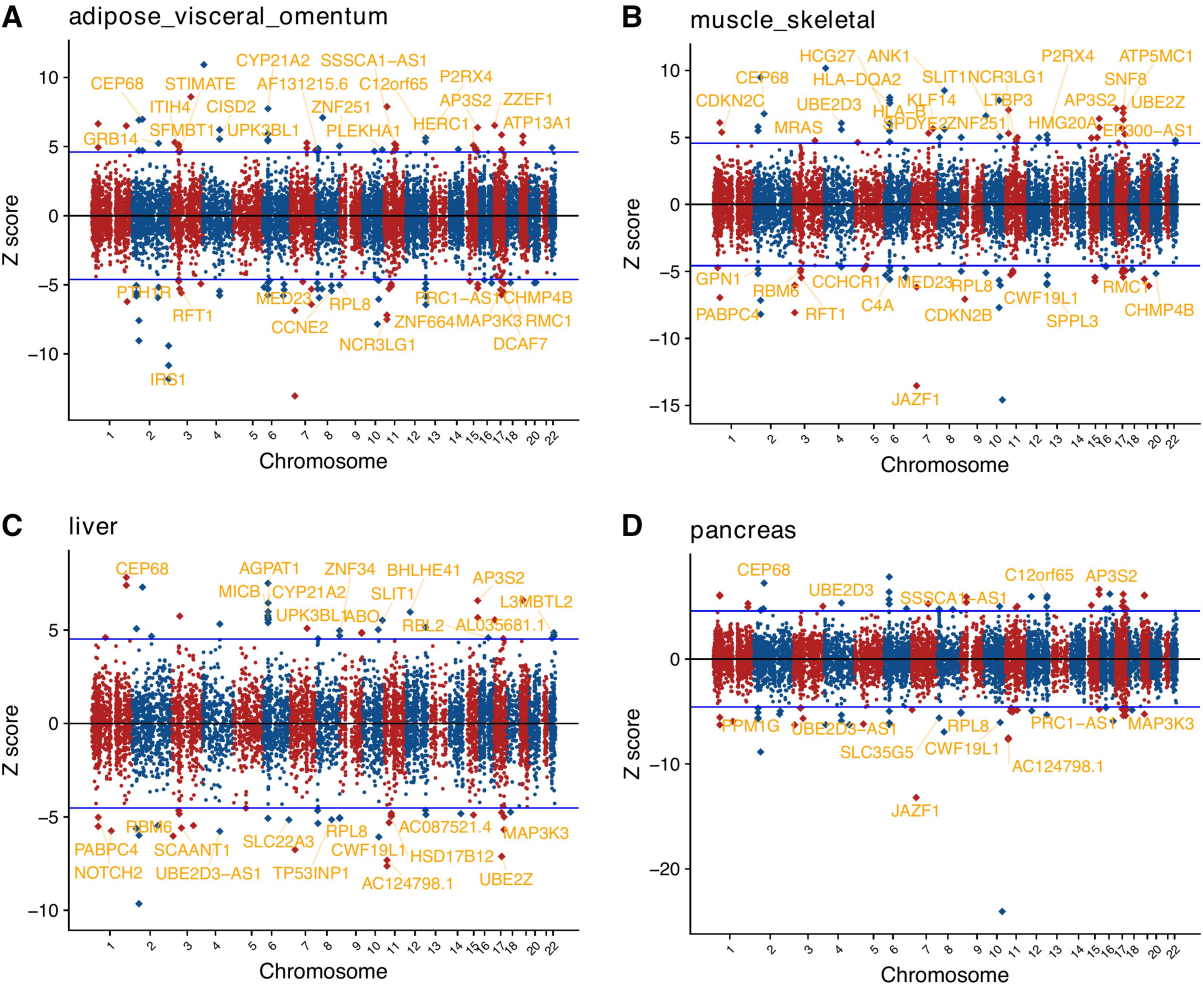
TWAS and colocalization analysis for T2D. Manhattan plots of TWAS results for T2D in visceral adipose (A), skeletal muscle (B), liver (C), and pancreas (D), respectively. The *Z*-scores for TWAS are shown. *Z*-score >0 indicates a gene for which higher genetically determined expression is associated with T2D. The labeled genes survived multiple testing corrections (over or below the blue lines) given the number of genes tested. Genes that further pass the significance threshold from the colocalization analysis are colored gold.

For T2D, 86 genes were identified by both TWAS and coloc/coloc-SuSiE in tissues examined (Fig. 3). We further investigated their associations with T2D in the Type 2 Diabetes Knowledge Portal (Costanzo *et al.* 2023). Among the 86 genes, 10 have been classified as potential T2D effectors, including *Insulin receptor substrate 1* (*IRS1*), a gene central in insulin signal transmission (Sun *et al.* 1992). We also identified T2D-associated genes *PTH1R* and *NOTCH2*, both druggable. *PTH1R* showed a negative effect on T2D in visceral adipose tissue. Teriparatide (TPTD), abaloparatide agonize *PTH1R*, and TPTD may improve glucose control in select populations (Mazziotti *et al.* 2014). Similarly, *NOTCH2* showed a negative effect on T2D in both liver and visceral adipose tissue (though drugs targeting NOTCH2 like nirogacestat and rg-4733 are inhibitory).

### 3.2 Multitarget drug repositioning using TReD

Next, we applied TReD to prioritize potential drug repurposing candidates for COVID-19 and T2D. We sought to identify significant reversal effects from the cellular drug response profiles on the population-level disease signatures at transcriptome level (see Section 2.7). In addition to four TWAS-derived disease signatures, we included three DGE-based disease signatures [for COVID-19: ALV (alveolar), EXP (expansion), BALF (bronchoalveolar lavage fluid); for T2D: islets, myoblasts, myotubes; Section 2.7]. Rules for prioritizing drugs are discussed in Section 2.7. Briefly, we defined a metric called reversal distance (*rd*) as a metric of how a drug may counteract a disease signature (defined by cellular transcriptomic response to the drug). For a drug to be considered a potential candidate, its reversal distance must be two or more standard deviations away from the mean *rd* and show nominal significance in a permutation test. As we observed highly consistent results with and without accounting for gene correlations (*r* = 0.8), the regular permutation results without accounting for gene correlations was presented. To ensure robustness, we further required consistent results across technical LINCS replicates, and at least one drug response profile analysis (permutation *P*-value) for the given drug must pass Bonferroni correction.

**Figure 4.**
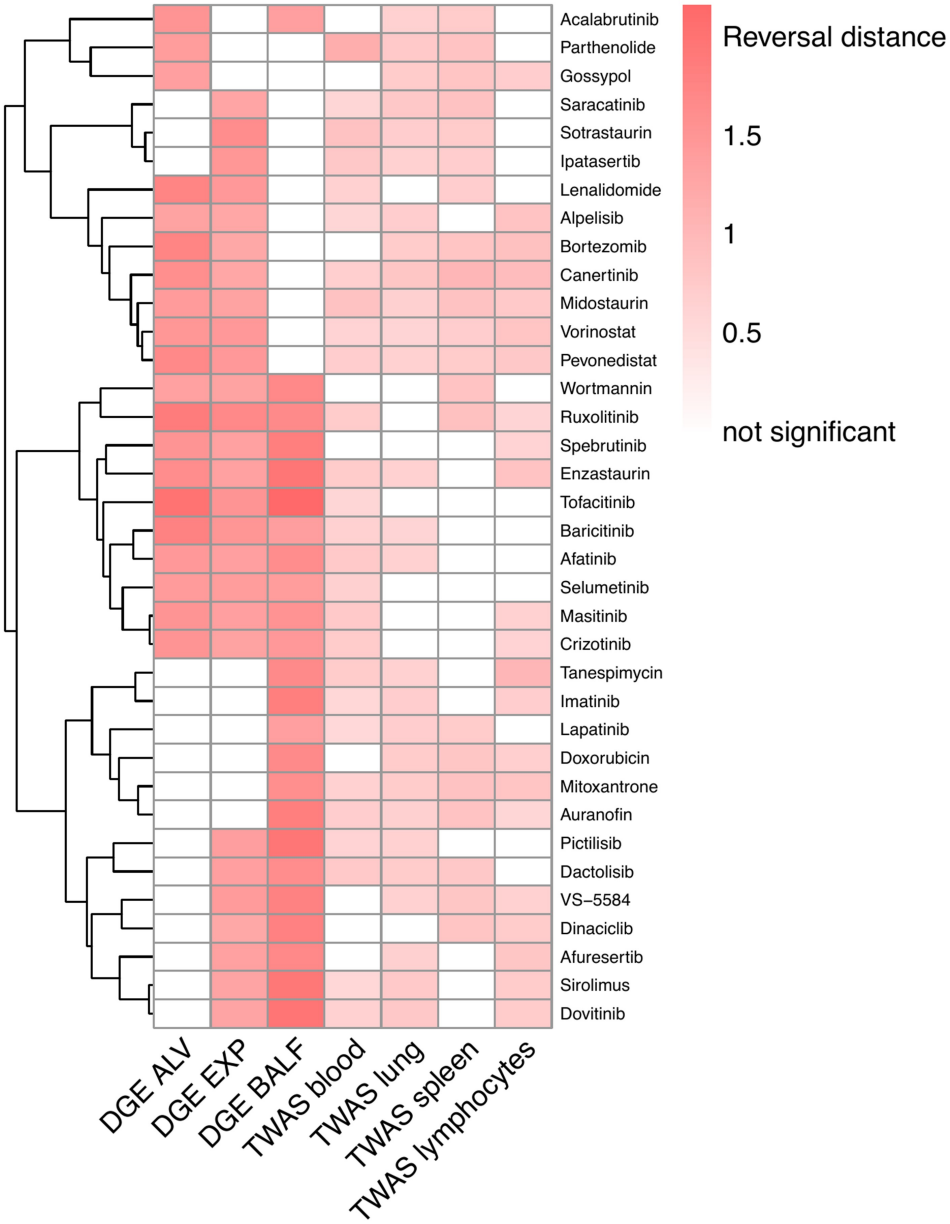
Drug repositioning candidates with potential reversal effect on COVID-19 disease signatures from DGE and TWAS. In total, 36 drugs were found by our Transcriptome-informed Reversal Distance (TReD) framework (see Section 2.7) to have a potential reversal effect on COVID-19 as indicated by at least four of the seven tested transcriptome-based disease signatures (*x*-axis). The heatmap shows the reversal distance for each drug response profile/disease signature pair. Blank cells denote “not significant” in the permutation test (the information on significance can be found in Table 5, available as supplementary data at *Bioinformatics* online). Hierarchical clustering was performed on the drug response profile (*y*-axis). The source data for the disease signatures, including TWAS (four tissue/cell types) and DGE (ALV, EXP, and BALF), are described in Section 2.

For COVID-19, we identified 707 drugs showing nominal significance in any of the seven disease signatures. The reversal distance values and permutation test *P*-values (both before and after adjustment) for all drugs can be found in Table 4, available as supplementary data at *Bioinformatics* online. For perturbations without repeated LINCS experiments, a consistent result in a confirmatory cell line is required to support the candidate drug response profile. Additionally, we highlighte*d* drugs that exhibited a reverse effect in at least four disease signatures. Lastly, we prioritized 36 drugs for COVID-19 that show significance (*P*_adj_<.05) in at least one drug response profile (Fig. 4). Table 1 provides an overview of the 36 drugs, including the reversal distance across the different disease signatures and whether there is any prior evidence linking to COVID-19 and/or clinical studies for the treatment of COVID-19. Notably, nearly 70% of the drugs (25/36) are reported related to COVID-19, and seven drugs have ongoing/completed clinical trials (Table 1).

**Table 1.** DrugBank annotations for drugs with significant reversal effects on at least four disease signatures for COVID-19.

Drug hit	Description	Reversal distance for each of the disease signatures							Evidence for COVID-19	Evidence for clinical trials
		DGE			TWAS					
		ALV	EXP	BALF	Blood	Lung	Spleen	Lymphocytes		
Wortmannin	PI3Ks inhibitor	1.35	1.311	1.699	n.s.	n.s.	0.829	n.s.	Yes (Jiang *et al.* 2022)	No
VS-5584	Antineoplastic	n.s.	1.426	1.784	n.s.	0.644	0.794	0.64	Yes (Bharadwaj *et al.* 2022)	No
Vorinostat	Antineoplastic	1.465	1.458	n.s.	0.632	0.597	0.7	0.813	Yes (Sinha *et al.* 2020)	No
Tofacitinib	Antineoplastic and immunomodulating	1.991	1.518	2.159	0.583	n.s.	n.s.	n.s.	Yes (Guimarães *et al.* 2021)	Yes
Tanespimycin	Antitumor antibiotics	n.s.	n.s.	1.665	0.74	0.653	n.s.	1.026	No	No
Spebrutinib	Immunomodulatory	1.507	1.333	1.828	n.s.	n.s.	n.s.	0.634	Yes (Kaliamurthi *et al.* 2021)	No
Sotrastaurin	Immunosuppressant	n.s.	1.625	n.s.	0.855	0.72	0.727	n.s.	Yes (Huang *et al.* 2022)	No
Sirolimus	Immunosuppressant	1.323	1.299	1.938	0.555	0.743	n.s.	0.733	Yes (Bischof *et al.* 2021)	Yes
Selumetinib	Antineoplastic	1.426	1.406	1.411	0.667	n.s.	n.s.	n.s.	Yes (Xie *et al.* 2022)	No
Ruxolitinib	Antineoplastic	1.871	1.685	1.666	0.722	n.s.	0.878	0.592	Yes (Agarwal *et al.* 2020)	Yes
Pictilisib	PI3K inhibitors	n.s.	1.377	1.944	0.615	0.636	n.s.	n.s.	No	No
Pevonedistat	Antineoplastic	1.686	1.45	n.s.	0.709	0.65	0.732	0.785	No	No
Parthenolide	Anti-inflammatory	1.403	n.s.	n.s.	1.138	0.753	0.841	n.s.	Yes (Bahrami *et al.* 2020)	No
Mitoxantrone	Chemotherapy drugs	n.s.	1.242	1.599	0.648	0.729	0.847	0.821	Yes (Preet *et al.* 2022)	No
Midostaurin	Antineoplastic	1.438	1.325	n.s.	0.863	0.672	0.853	0.748	No	No
Masitinib	Antineoplastic and immunomodulating	1.511	1.372	1.529	0.75	n.s.	n.s.	0.639	Yes (Drayman *et al.* 2021)	Yes
Lenalidomide	Antineoplastic and immunomodulating	1.75	1.441	n.s.	0.646	n.s.	0.714	n.s.	Yes (Von Metzler *et al.* 2022)	No
Lapatinib	Antineoplastic	n.s.	n.s.	1.357	0.541	0.709	0.733	n.s.	Yes (Saul *et al.* 2023)	No
Ipatasertib	Antineoplastic	n.s.	1.478	n.s.	0.768	0.654	0.708	n.s.	No	No
Imatinib	Antineoplastic	n.s.	n.s.	1.814	0.552	0.7	n.s.	0.719	Yes (Van De Veerdonk *et al*. 2022)	Yes
Gossypol	Polyphenolic compounds	1.374	n.s.	n.s.	n.s.	0.738	0.824	0.711	Yes (Wang *et al.* 2021)	No
Enzastaurin	Antineoplastic	1.616	1.346	1.957	0.738	0.642	n.s.	0.84	Yes (Huang *et al.* 2022)	No
Doxorubicin	Chemotherapy drugs	n.s.	n.s.	1.66	n.s.	0.73	0.798	0.685	Yes (Sajid Jamal *et al.* 2022)	No
Dovitinib	Multitargeted kinase inhibitor	n.s.	1.304	1.964	0.652	0.782	n.s.	0.737	No	No
Dinaciclib	Antineoplastic	n.s.	1.236	1.8	n.s.	n.s.	0.816	0.738	Yes (Ahmad *et al.* 2023)	No
Dactolisib	Antineoplastic	n.s.	1.363	1.629	0.761	0.732	0.772	n.s.	Yes (Tomazou *et al.* 2021)	No
Crizotinib	Antineoplastic	1.513	1.318	1.459	0.72	n.s.	n.s.	0.625	Yes (Chen *et al.* 2021)	No
Canertinib	Antineoplastic	1.613	1.253	n.s.	0.687	0.801	1.029	0.957	No	No
Bortezomib	Antineoplastic	1.766	1.241	n.s.	n.s.	0.734	0.819	0.883	Yes (Li *et al.* 2022)	No
Baricitinib	Immunosuppressant	1.767	1.493	1.393	0.633	0.594	n.s.	n.s.	Yes (Kalil *et al.* 2021)	Yes
Auranofin	Anti-inflammatory and antirheumatic	n.s.	n.s.	1.815	0.715	0.664	0.844	0.588	Yes (Biji *et al.* 2021)	No
Alpelisib	Antineoplastic	1.319	1.249	n.s.	0.582	0.711	n.s.	0.84	No	No
Afuresertib	Akt inhibitor	n.s.	1.334	1.68	n.s.	0.669	n.s.	0.794	No	No
Afatinib	Antineoplastic	1.442	1.369	1.628	0.744	0.641	n.s.	n.s.	No	No
Acalabrutinib	Antineoplastic	1.526	n.s.	1.355	n.s.	0.65	0.722	n.s.	Yes (Roschewski *et al.* 2020)	Yes
Saracatinib	Antineoplastic	n.s.	1.279	n.s.	0.576	0.771	0.859	n.s.	No	No

The estimated reversal distance is shown. n.s. denotes not significant in the permutation test. The source data for the disease signatures, including TWAS (four tissue/cell types) and DGE (ALV, EXP, and BALF), are described in Sections 2.3 and 2.4.

**Table 2.** DrugBank annotations for drugs with significant reversal effects on two disease signatures for T2D.

Drug hit	Description	Reversal distance for each of the disease signatures							Evidence for COVID-19	Evidence for clinical trials
		DGE			TWAS					
		Islets	Myoblasts	Myotubes	Adipose	Muscle	Liver	Pancreas		
AMG-232	Antineoplastic	n.s.	2.452	n.s.	n.s	n.s.	n.s.	0.643	No	No
Batimastat	Antineoplastic	n.s.	2.063	n.s.	n.s.	n.s.	n.s.	0.715	Yes (Nose *et al.* 2003)	No
Imatinib	Tk inhibitor	n.s.	2.094	n.s.	n.s.	n.s.	0.666	n.s.	Yes (Veneri *et al.* 2005)	No
Pimozide	Antipsychotic	n.s.	1.692	n.s.	n.s.	n.s.	0.83	n.s.	Yes (Al Batran *et al.* 2020)	No
Bentazepam	Antipsychotic	n.s.	1.448	n.s.	n.s.	0.586	n.s.	n.s.	No	No
Guanethidine	Antihypertensive drug	n.s.	1.463	n.s.	n.s.	0.652	n.s.	n.s.	No	No
Cabozantinib	Antineoplastic	n.s.	1.657	n.s.	n.s.	n.s.	n.s.	0.634	Yes (Ye *et al.* 2022)	No
Metoprolol	Antiarrhythmic	n.s.	1.371	n.s.	n.s.	n.s.	n.s.	0.735	Yes (Sharma *et al.* 2008)	No
AMD-070	CXCR4 antagonist	1.231	n.s.	n.s.	n.s.	n.s.	0.645	n.s.	No	No
AZD-1480	JAK inhibitor	1.353	n.s.	n.s.	0.666	n.s.	n.s	n.s	No	No
Prochlorperazine	Antipsychotic	1.202	n.s.	n.s.	n.s.	n.s.	n.s.	0.652	No	No
CTS-1027	Amines	n.s.	1.556	n.s.	n.s.	n.s.	n.s.	0.631	No	No
Podofilox	Antimitotic	1.231	n.s.	n.s.	n.s.	0.588	0.657	n.s.	No	No
Palbociclib	Antineoplastic	0.789	2.057	n.s.	n.s.	n.s.	0.598	n.s.	No	No
Ponatinib	Antineoplastic	n.s.	2.103	n.s.	0.848	n.s.	n.s.	0.699	No	No
Tamatinib	Syk inhibitor	n.s.	1.922	n.s.	n.s.	n.s.	0.584	0.688	No	No

The estimated reversal distance is shown. n.s. denotes not significant in the permutation test. The source data for the disease signatures, including TWAS (four tissue/cell types) and DGE (islets, myoblasts, and myotubes), are described in Sections 2.3 and 2.4.

**Table 3.** DrugBank annotations for drugs with significant reversal effects on at least four disease signatures for AD.

Drug hit	Description	Significant reversal effects on each of the AD TWAS signatures								Evidence for AD		Evidence for clinical trials
		Amygdala	Cortex BA24	Caudate	Cortex	Hippo-campus	Hypotha-lamus	Nucleus-accumbens	Cortex BA9			
Olanzapine	Antagonists	Sig	n.s.	Sig	Sig	Sig	Sig	Sig	Sig	Yes (Johnson *et al.* 2022)	Yes	
Tianeptine	Antidepressive	Sig	n.s.	n.s.	Sig	Sig	n.s.	Sig	Sig	Yes	No	
Saracatinib	TK inhibitor	Sig	Sig	Sig	Sig	Sig	Sig	n.s.	Sig	Yes (Nygaard *et al.* 2015)	Yes	
Tanespimycin	HSP90 inhibitors	Sig	Sig	Sig	n.s.	Sig	Sig	Sig	Sig	Yes (Chen *et al.* 2014)	No	
Tofacitinib	JAK inhibitor	Sig	Sig	Sig	n.s.	Sig	n.s.	Sig	n.s.	Yes (Rodriguez *et al.* 2021)	Yes	
Oxiracetam	Nootropic	n.s.	n.s.	Sig	Sig	Sig	Sig	Sig	Sig	Yes (Zhang *et al.* 2020)	No	
Alvocidib	CDK inhibitor	Sig	n.s.	Sig	Sig	Sig	Sig	Sig	n.s.	Yes (Leggio *et al.* 2016)	No	
Dacomitinib	Antineoplastic	Sig	n.s.	n.s.	Sig	n.s.	n.s.	Sig	Sig	No	No	
Marbofloxacin	Anti-bacterial	n.s.	n.s.	Sig	n.s.	Sig	Sig	Sig	n.s.	No	No	
Motesanib	Enzyme inhibitors	n.s.	n.s.	Sig	n.s.	Sig	Sig	Sig	n.s.	No	No	
OSI-930	Sulfur	Sig	n.s.	Sig	Sig	Sig	n.s.	Sig	n.s.	No	No	

The estimated reversal distance is shown. n.s. denotes not significant in the permutation test. The source data for the disease signatures are described in Sections 2.8.

Of note, over 40% of candidates are immunomodulatory drugs, especially JAK inhibitors. Baricitinib, significantly reversing five disease signatures (Fig. 6), is a JAK1/2 and TYK2 inhibitor approved for COVID-19 therapy in 2022. Ruxolitinib significantly reversed six disease signatures (Fig. 6), with a variety of reports supporting divergent clinical efficacy (Han *et al.* 2022, Rein *et al.* 2022). Analogously, enzastaurin—investigational for treatment of glioblastoma multiforme via selectively inhibition of PKC-beta and other kinases involved in cell proliferation and survival—appeared effective in reversing six disease signatures (Fig. 6**)**. Of note, SARS-CoV can activate the PKC members, and inhibitors of protein kinase C family members, including enzastaurin, may decrease SARS-CoV-2 replication *in vitro* (Huang *et al.* 2022).

**Figure 5.**
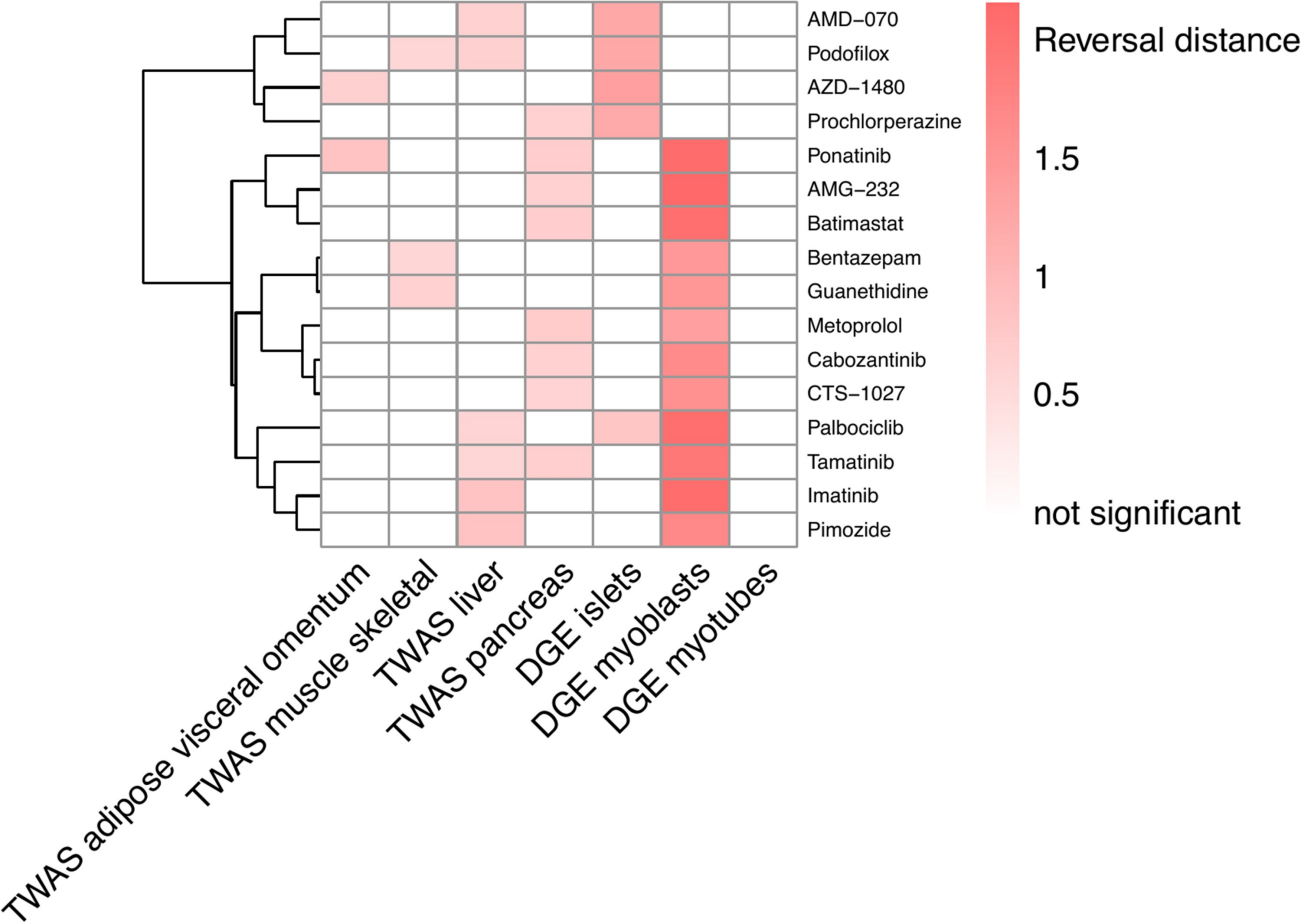
Drug repositioning candidates with potential reversal effect on T2D disease signatures from DGE and TWAS. In total, 16 drugs were found by our Transcriptome-informed Reversal Distance (TReD) framework (see Section 2.7) to have a potential reversal effect on T2D as indicated by two of the seven tested transcriptome-based disease signatures (*x*-axis). The heatmap shows the reversal distance for each drug response profile/disease signature pair. Blank cells denote “not significant” in the permutation test (the information on significance can be found in Table 6, available as supplementary data at *Bioinformatics* online). Hierarchical clustering was performed on the drug response profile (*y*-axis). The source data for the disease signatures, including TWAS (four tissue/cell types) and DGE, are described in Section 2.

For T2D, we identified 78 drugs/compounds as potential candidates for at least two T2D disease signatures (one TWAS and one DGE). The reversal distance values and permutation test *P*-values (both before and after adjustment) for all drugs can be found in Supplemental Table 4, available as supplementary data at *Bioinformatics* online. We further filtered out 62 drugs/compounds without any drug response profile passing multiple comparison. This resulted in a total of 16 drugs/compounds (Table 2, Fig. 5). Five of the drugs/compounds have been previously reported as potential options for the prevention or treatment of T2D. For example, imatinib (notably elevated reversal distance) has been shown to improve insulin sensitivity and maintain glucose homeostasis potentially via effects on endoplasmic reticula stress (Han *et al.* 2009, Fig. 7). Pimozide also showed a significant reversal effect and may alleviate hyperglycemia in diet-induced obesity through inhibition of skeletal muscle ketone oxidation (Al Batran *et al.* 2020,  Fig. 7). Batimastat demonstrated a significant reversal effect, with potential roles in T2D-related complications as a matrix metalloproteinase inhibitor (Ramnath *et al.* 2014, Fig. 7). Collectively, these results suggest pathophysiologic plausibility of TReD-defined targets in T2D.

**Figure 6.**
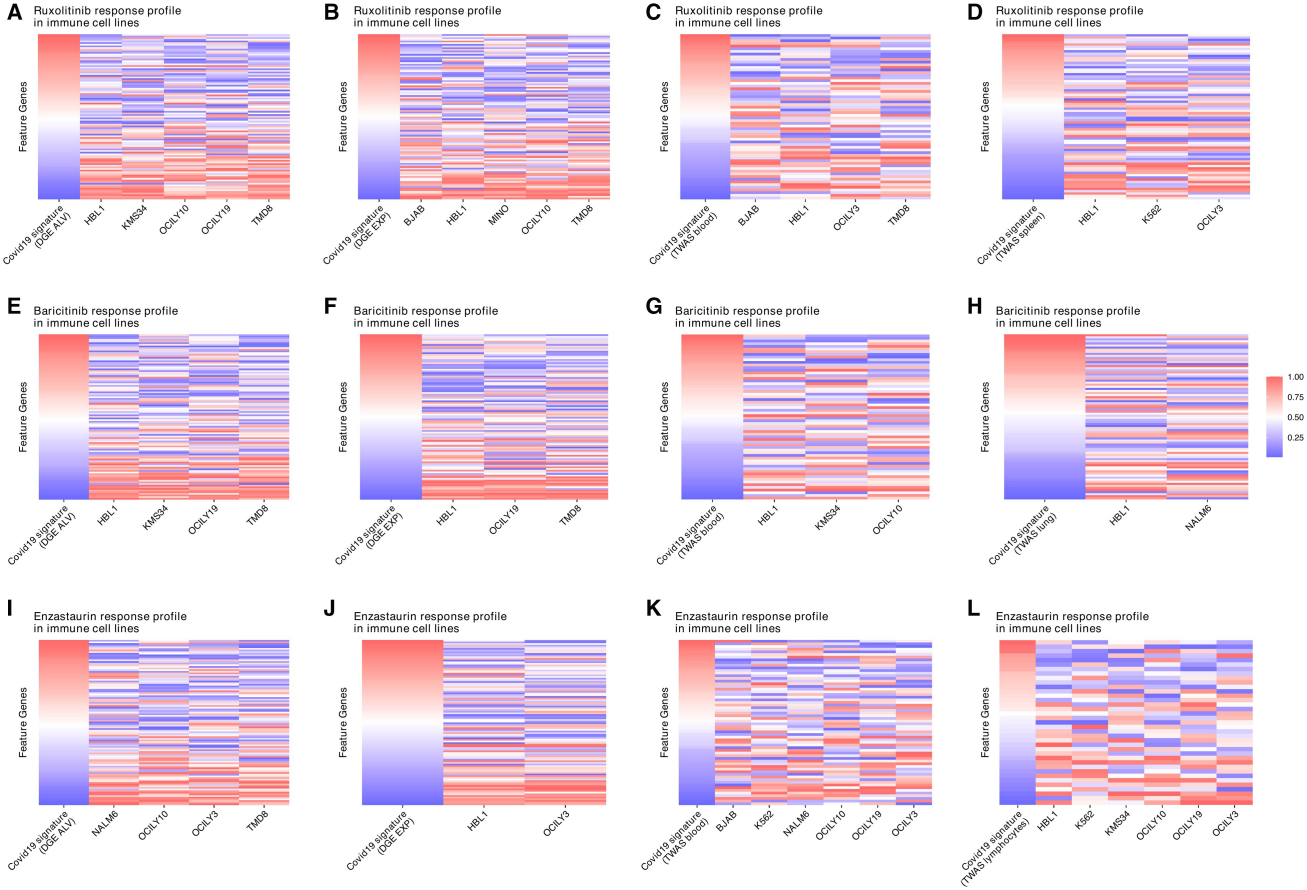
TReD-identified relationships between COVID-19 signatures and drug response profiles. The potential reversal effects of Ruxolitinib (A–D), Baricitinib (E–H), and Enzastaurin (I–L), which were identified against at least four disease signatures of COVID-19, are visualized using a heatmap. Taking (A) as an example, the first column in the heatmap represents differential gene expression (DGE) from the ALV gene expression disease signature ranked by fold change. Red and blue denote higher and lower expressed in affected samples, respectively. The remaining columns represent gene expression changes after Ruxolitinib treatment in immune cells. For drugs with multiple significant profiles, the drug response profiles (e.g. different duration after treatment) with the largest reversal distance are presented. The source data for the disease signatures, including TWAS (four tissue/cell types) and DGE (ALV and EXP), are described in Sections 2.3 and 2.4.

**Figure 7.**
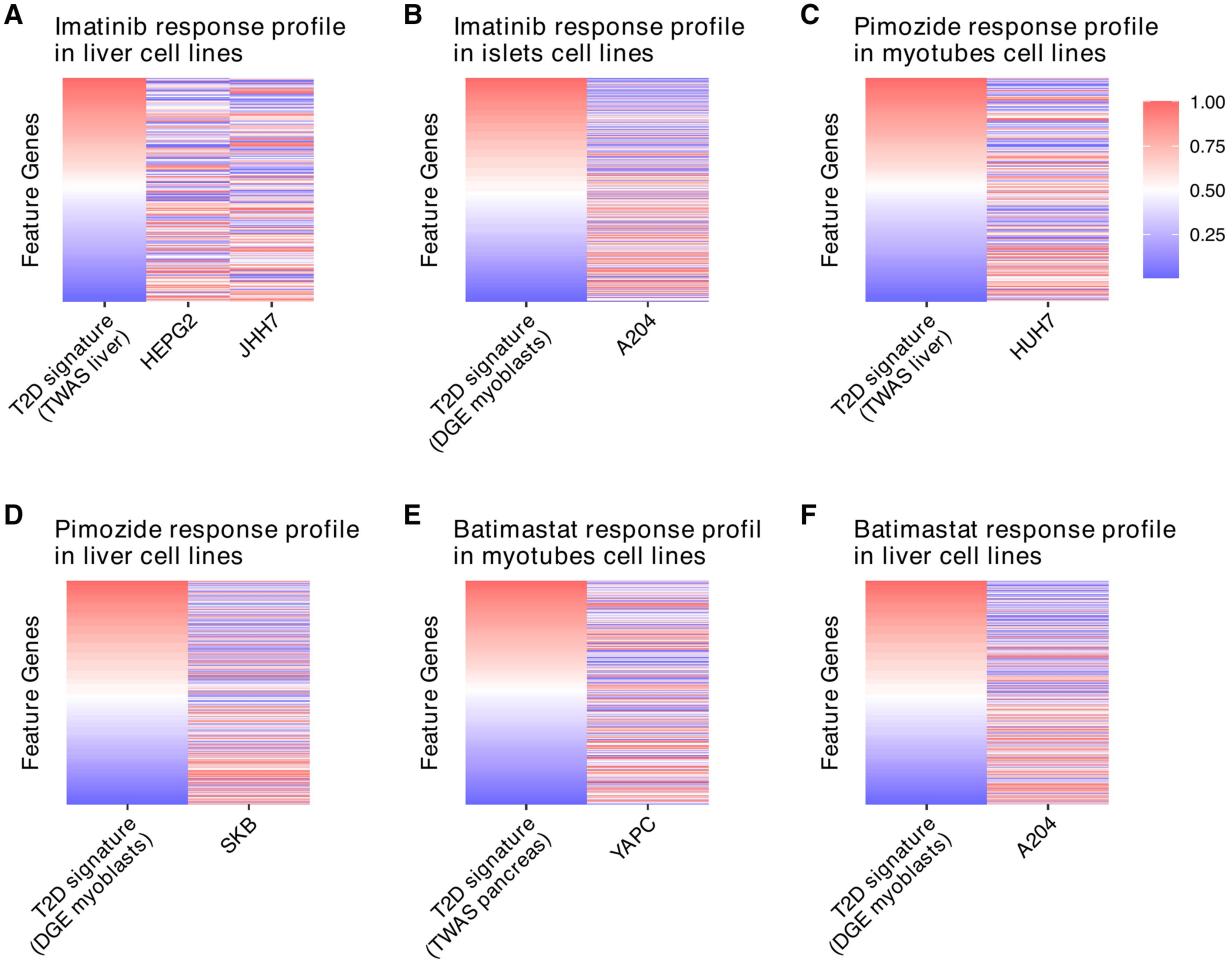
TReD-identified relationships between T2D signatures and drug response profiles. The potential reversal effects of Imatinib (A, B), Pimozide (C, D), and Batimastat (E, F), which were identified against two disease signatures of T2D, are visualized using a heatmap. Taking (A) as an example, the first column in the heatmap represents the TWAS-derived disease signature in liver ranked by fold change. Red and blue denote higher and lower expressed in affected samples, respectively. The remaining columns represent gene expression changes after drug treatment in liver cells. For drugs with multiple significant profiles (e.g. different duration after treatment), the drug response profiles with the largest reversal distance are presented. The source data for the disease signatures, including TWAS (four tissue/cell types) and DGE, are described in Sections 2.3 and 2.4.

### 3.3 Further validation of TReD in AD

For AD, we selected 11 compounds that showed nominal significance in at least four TWAS signatures and had at least one drug response profile passing multiple comparison correction (Table 3). The reversal distance values and permutation test *P*-values (both before and after adjustment) for all drugs can be found in Table 14, available as supplementary data at *Bioinformatics* online. Several candidate compounds were prioritized with known or emerging therapeutic relevance in AD. Notably, Olanzapine, already used clinically for behavioral symptoms in AD, and Tianeptine, with neuroprotective effects, were prioritized. Saracatinib has entered clinical trials for AD, while Tanespimycin and Tofacitinib target AD-relevant pathways like protein aggregation and inflammation. Oxiracetam, a cognitive enhancer, also emerged. Details of the 11 drugs are provided in Table 3.

### 3.4 Comparison of TReD with the KS statistic-based pattern matching approach

For COVID-19, eight compounds were identified by KS-based method, but only one had been previously reported in the literature as related to COVID-19 treatment, and none had entered clinical trials (Table 15, available as supplementary data at *Bioinformatics* online). For T2D, 18 drugs were identified through screening, among which three had been previously reported in the literature as related to T2D treatment, but none had entered clinical trials (Table 16, available as supplementary data at *Bioinformatics* online).

We further quantitatively compared TReD with the KS-based method. Among KS-identified compounds, 12.5% (COVID-19) and 17% (T2D) had literature support, with none in clinical trials. In contrast, TReD achieved higher validation rates—70% (COVID-19) and 31% (T2D)—with 7 of 36 COVID-19 compounds having entered clinical trials.

## 4 Discussion

Drug development pipelines have traditionally relied on high-throughput rational or biological synthesis and screening approaches. Recent high-dimensional genomic and transcriptional approaches have yielded a path to prioritize potential targets for directed study (Sass *et al.* 2024). However powerful, traditional genomic approaches (e.g. TWAS) alone only identify potential pathways to target, without taking advantage of the large bank of information on druggability and cellular responses to drugs already in existence. Here, we developed a framework to identify potential drug repurposing candidates by combining population-level disease signatures via human genetics (TWAS/DGE) and cellular responses to drugs at the transcriptional level (TReD). These approaches were successfully applied to COVID-19 and T2D, identifying known (e.g. baricitinib/TYK2) and novel drug–disease combinations. To further evaluate the translational potential of the TReD framework, we applied it to AD. The findings in AD demonstrate the broader applicability of TReD and its capacity to prioritize drugs with therapeutic potential across diverse diseases. Additionally, we applied TReD and the KS-based method to the same dataset for a comparative analysis, and observed that TReD consistently outperformed the KS-based method in identifying a literature-supported and clinically investigated drug.

We also investigated the drugs that are positively correlated with the disease signatures of COVID-19. A few such top-ranked drugs [including testosterone (Yassin *et al.* 2023) and otamixaban (Hempel *et al.* 2021)] were also supported by published studies as potential treatment options. This scenario may arise, for example, when a disease signature targeted by such a drug lies in a coexpression network with another disease signature whose net reversal is a potential therapeutic option. This result indicates that, in reality, the biological system operates in a much more complex fashion. These findings highlight the importance of further experimental validation to elucidate the precise mechanisms and assess the therapeutic potential of such candidate drugs.

The strengths of our framework stem from several key aspects. Firstly, the amalgamation of single-target and multiple-target approaches enables a more comprehensive therapeutic search, and this is particularly valuable for addressing complex diseases involving multiple biomolecules or mechanisms. Secondly, the framework incorporates two complementary methods (DGE and TWAS) as population-level sources for disease signatures. Although TWAS may be underpowered with a limited sample size, a critical feature of such a genetics-anchored method ensures that it identifies potentially regulatory or candidate causal genes for the disease, rather than genes altered by the disease. In contrast, DGE is prone to identify the transcriptomic consequences of the disease; nevertheless, it remains well-powered even with limited sample sizes. The combination of these two methods should enrich for upstream mediators and molecular consequences, facilitating the discovery of new drug indications or the repositioning of existing drugs. Thirdly, the use of the reversal distance measurement in our study capitalizes on available (and growing) information from transcriptome-level disease-associated gene features and drug response profiles, with biologically relevant interpretation of an effective therapeutic effect on disease. This differs from previous and, we would argue, less interpretable methods, such as those that have relied solely on a nonparametric KS statistic, focusing on the maximum difference in the relative ranks of the upregulated and downregulated genes. Fourthly, we leveraged the latest CMAP LINCS 2020 data which contains over 3 million gene expression profiles with >80 000 perturbations and >200 cell lines affecting over 12 328 genes, providing a comprehensive candidate pool for drug repositioning. While our study focused on COVID-19 and T2D, our framework is highly generalizable and can be implemented to identify drug repositioning candidates for other diseases. In the future, if the LINCS/CMAP publishes the perturbation signatures of drug combinations, TReD can also be extended to finding drug combinations for the target disease.

While encouraging as a first attempt to integrate TWAS and cellular response profiles, TReD has a key limitation: this approach is dependent on comprehensive curation of drug response profiles over a wide range of drug targets, cell types, and disease conditions. Indeed, while we were broad in scope for inclusion of curated data for these elements, the cellular response profiles were obtained primarily in cancer cell lines, which may not necessarily phenocopy the cell types and biological context of interest for the condition under study (e.g. COVID-19). In addition, we recognize that most medications here are necessarily anticancer therapeutics, which may not be clinically translatable to other broad conditions (e.g. T2D) due to off-target (side) effects. Finally, other “omes” beyond transcriptional responses are likely relevant (e.g. protein expression, noncoding RNA) that may encode *trans*-organ signaling pathways and a broad cell atlas relevant to multisystem disorders (e.g. T2D). Our study does not consider the complex issues of drug–target interactions and drug–drug interaction, and consideration of *in vivo* pharmacokinetics, pharmacodynamics, and substrate processing are critical. The solution to these limitations is either a strength of older drug discovery approaches—broad, rapid screening functional assays across cellular phenotypes and broad synthetic or derivatized chemical libraries or biologics for screening [e.g. rapid lentiviral CRISPR screening (Chaffin *et al.* 2022)]—or a strength of ongoing drug development pipelines (Phase 0 and animal system studies). The role for TReD in this pipeline is to hone the potential space of therapeutics and provide a multidimensional platform to integrate across molecular genetic information to improve drug discovery efficiency.

Looking forward, integrating deep learning techniques into the TReD framework may offer a powerful solution to some of these limitations. For example, graph neural networks can model complex drug–gene–disease relationships, while attention-based models or multimodal neural networks can integrate heterogeneous omics data to better capture the intricate biological context. These methods may also allow for improved generalization across different cell types or conditions and more robust feature learning in data-limited scenarios. Thus, we envision that coupling deep learning with the TReD platform could substantially enhance its predictive performance, interpretability, and applicability in drug discovery and repositioning.

## 5 Conclusion

Integrating transcriptome-level profiles from cell-based drug response profiles and population-based disease signature studies, we developed a framework, TReD, to identify drug repurposing candidates. Deploying TReD in COVID-19 and T2D successfully identified both known and novel candidates that capture underlying pathobiological mechanisms important in each disease. These results suggest that computational approaches that unite molecular genetic information with cellular response profiles may be effective in drug repositioning across a wide array of disorders. Future work collating cell types, pharmacotherapies, and disease states is needed to fully realize the potential of this and analogous approaches.

## Supplementary Material

btaf498_Supplementary_Data

## Data Availability

Source code and datasets considered in this study are available at Github (https://github.com/zdangm/TReD). An archived snapshot is deposited at Zenodo (https://doi.org/10.5281/zenodo.16791909).

## Supplementary data

Conflict of interest: R.V.S. has equity ownership in Thryv Therapeutics and is a co-inventor on a patent for ex-RNAs signatures of cardiac remodeling and a pending patent on proteomic signatures of disease. E.R.G. has performed consulting for Thryv Therapeutics and is a co-inventor on disclosures and patents for molecular signatures of cardiovascular phenotypes and metabolic health, use of RNAs as therapeutics and biomarkers in disease, and methods in metabolomics. All authors read and approved the final manuscript.
