# Supplementary material for: Integrating population-level and cell-based signatures for drug repositioning
Source: Bioinformatics. 2025 Sep 10;41(10):btaf498. doi: 10.1093/bioinformatics/btaf498 (PMC12512135; doi:10.1093/bioinformatics/btaf498)
Supplement: btaf498_Supplementary_Data [file btaf498_supplementary_data.zip › Supplemental Material 1 (Figure S1-S3 and Table S15-S16).docx]

**Supplementary files**

**Supplemental Figure S1**: Application to identify drug repurposing candidates for COVID-19

**Supplemental Figure S2:** Venn diagram of TWAS significant and colocalization positive genes in four COVID-19 relevant tissues (blood, lung, lymphocytes, spleen)

**Supplemental Figure S1**: **Application to identify** **drug repurposing candidates for COVID-19** (a) GWAS summary statistics of COVID-19 severity and eQTL of four immune-related tissues were combined by PrediXcan and coloc/coloc-SuSiE, respectively. Genes that were both significant in TWAS after Bonferroni correction and exhibited positive colocalization signals (PP.H4 for coloc and coloc-SuSiE ≥ 0.85) were searched as potential drug targets. (b) Four TWAS results (*P_FDR_* < 0.05), three DGE for COVID-19 severity and cellular-level transcriptome drug profiles from CMAP LINCS 2020 were integrated by TReD to obtain drugs that reverse disease signature.


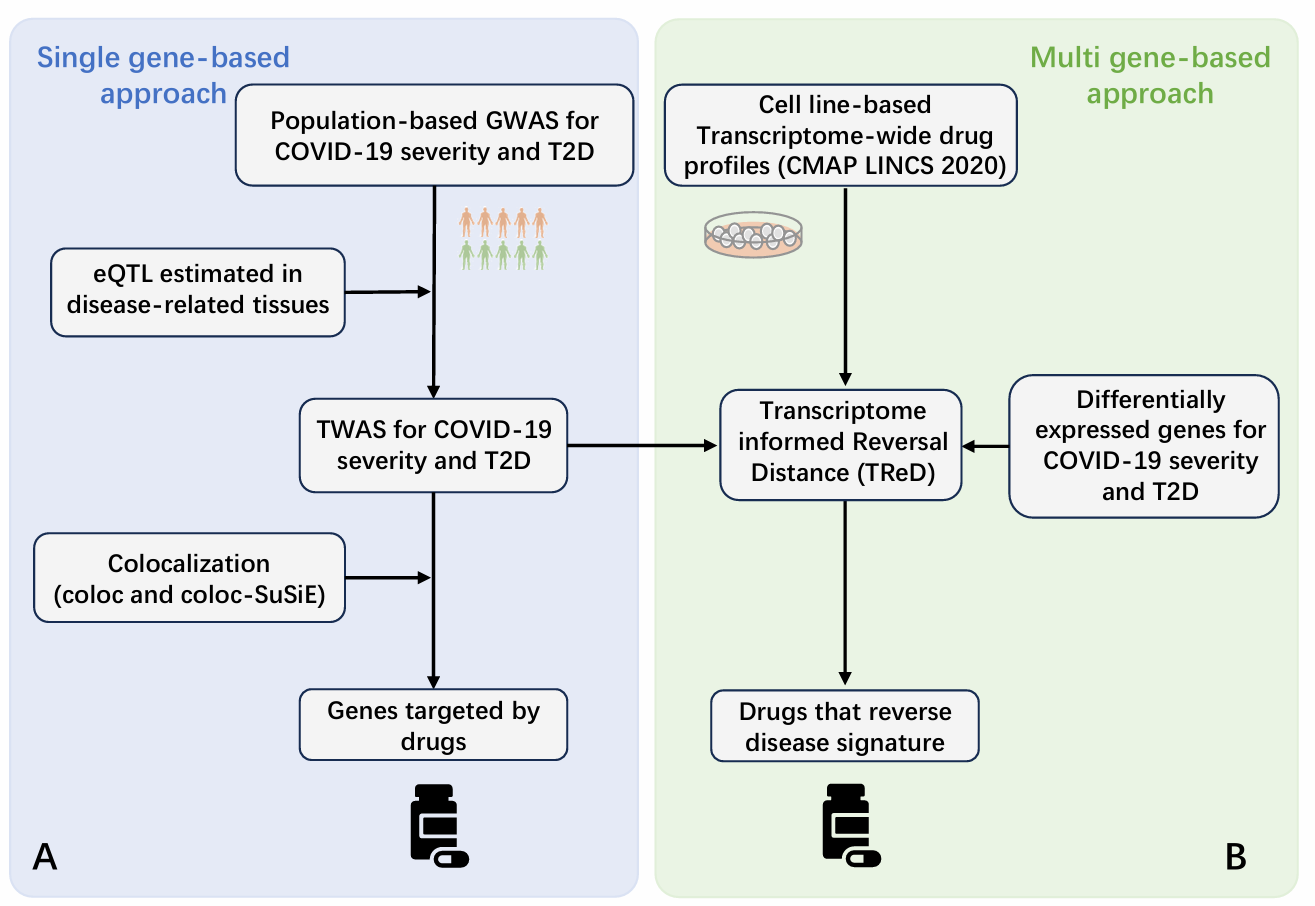


**Supplemental Figure S2: Venn diagram of TWAS significant and colocalization positive genes in four COVID-19 relevant tissues (blood, lung, lymphocytes, spleen)**. Blood had eleven genes, lungs had twelve, lymphocytes carried ten, and the spleen hosted twelve genes. In total, there were 28 distinct genes and eleven genes appeared in at least two tissues. Two genes overlapped among the four tissues.


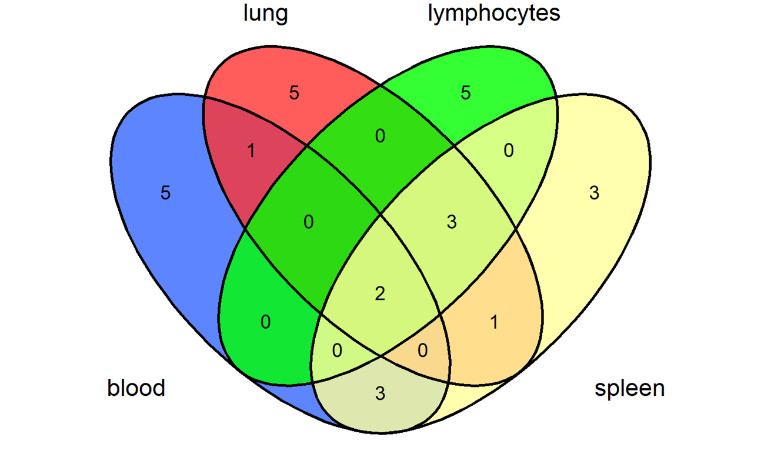


**Supplemental Figure S3:** **The workflow of TReD framework.**


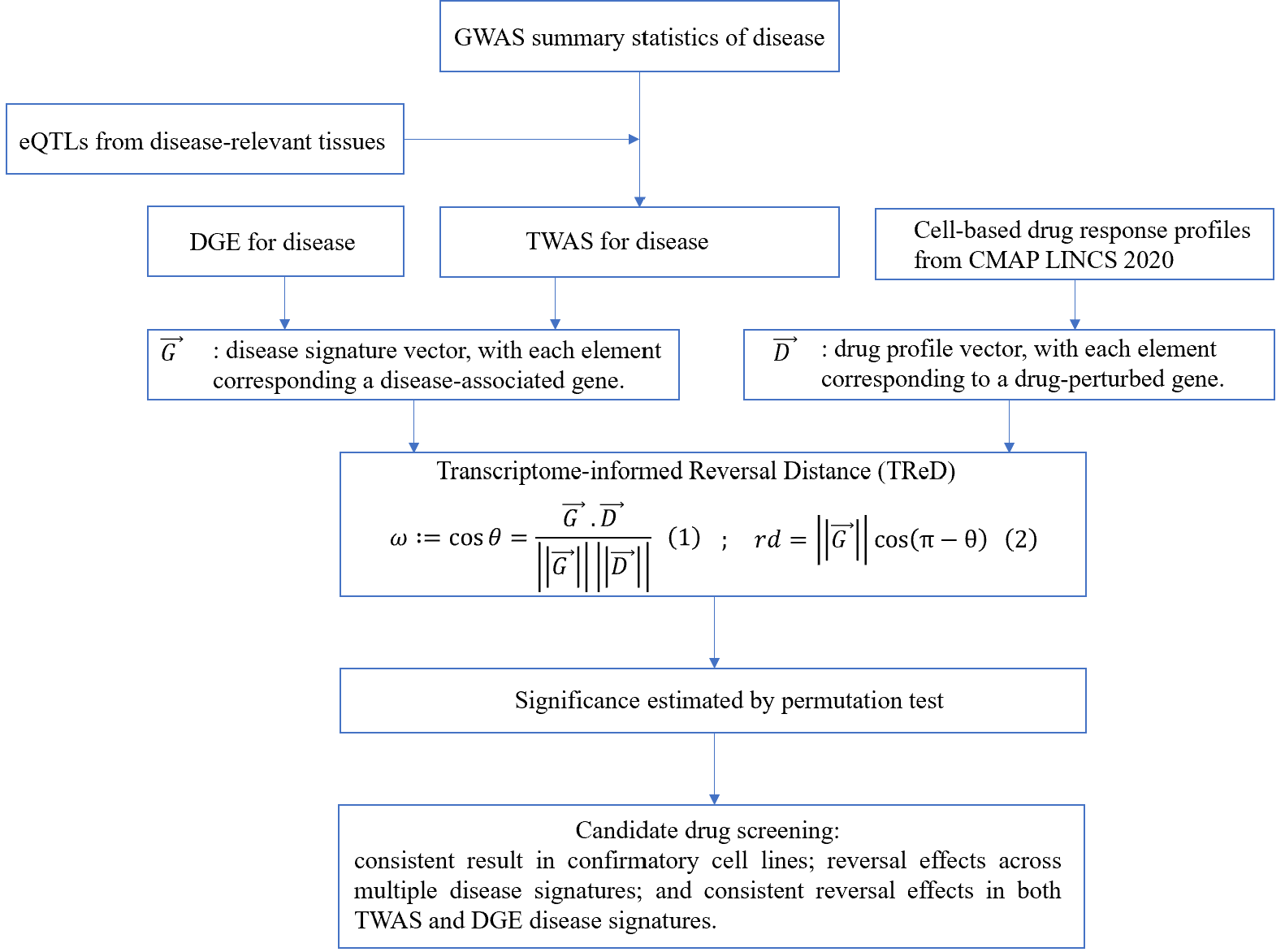


**Table S15. DrugBank annotations for drugs with significant reversal effects on at least four disease signatures for COVID-19.**

| Drug Hit | Description | Significant reversal effects on each of the disease signatures | | | | | | | Evidence  for  covid-19 | | Evidence  for  clinical trials |
| --- | --- | --- | --- | --- | --- | --- | --- | --- | --- | --- | --- |
|  |  | DGE  ALV | DGE  EXP | DGE  BALF | TWAS  blood | TWAS  lung | TWAS  spleen | TWAS  lymphocytes |  |  |  |
| Vorinostat | Antineoplastic | Sig | Sig | Sig | n.s | n.s. | n.s. | Sig | Yes ^[1]^ | No | |
| Triclabendazole | Anthelmintics | Sig | Sig | n.s. | n.s. | Sig | Sig | n.s. | No | No | |
| Tanespimycin | Amides | Sig | Sig | Sig | n.s. | Sig | n.s. | Sig | No | No | |
| Tamatinib | immunomodulatory | Sig | Sig | Sig | n.s. | n.s. | Sig | n.s. | No | No | |
| Pazopanib | Antineoplastic | Sig | Sig | Sig | n.s. | n.s. | Sig | n.s. | No | No | |
| Midostaurin | Antineoplastic | Sig | Sig | Sig | n.s. | Sig | n.s. | n.s. | No | No | |
| Idelalisib | Antineoplastic | Sig | Sig | Sig | n.s. | n.s. | Sig | n.s. | No | No | |
| Bortezomib | Antineoplastic | Sig | Sig | Sig | n.s. | n.s. | Sig | n.s. | No | No | |

[1] Tomazou M, Bourdakou MM, Minadakis G, et al. Multi-omics data integration and network-based analysis drives a multiplex drug repurposing approach to a shortlist of candidate drugs against COVID-19. Brief Bioinform. 2021 Nov 5;22(6):bbab114.

**Table S16. DrugBank annotations for drugs with significant reversal effects on two disease signatures for T2D.**

| Drug Hit | | Description | Significant reversal effects on the disease signatures | | | | | | | Evidence  for  T2D | | Evidence  for  clinical trials |
| --- | --- | --- | --- | --- | --- | --- | --- | --- | --- | --- | --- | --- |
|  |  |  | DGE  islets | DGE  myoblasts | DGE  myotubes | TWAS  Adipose | TWAS  Muscle | TWAS  Liver | TWAS  Pancreas |  |  |  |
| Ulixertinib | | Antineoplastic | Sig | n.s. | n.s. | n.s | n.s. | n.s. | Sig | No | No | |
| Tipranavir | | Anti-HIV Agents | Sig | n.s. | n.s. | n.s. | n.s. | n.s. | Sig | No | No | |
| Sotrastaurin | | Sotrastaurin | Sig | n.s. | n.s. | n.s. | n.s. | n.s. | Sig | No | No | |
| Oxybuprocaine | | Anesthetic | Sig | n.s. | n.s. | n.s. | n.s. | n.s. | Sig | No | No | |
| m-Chlorophenylpiperazine | | Antidepressive | Sig | n.s. | n.s. | n.s. | n.s. | n.s. | Sig | No | No | |
| LY-294002 | Enzyme Inhibitors | | n.s. | Sig | n.s. | n.s. | Sig | n.s. | n.s. | No | No | |
| Lisinopril | | ACE Inhibitors | Sig | n.s. | n.s. | n.s. | n.s. | n.s. | Sig | No | No | |
| Lesinurad | | Antigout | Sig | n.s. | n.s. | n.s. | n.s. | n.s. | Sig | No | No | |
| Indiplon | GABA Agents | | Sig | n.s. | n.s. | n.s. | n.s. | n.s. | Sig | No | No | |
| Geldanamycin | Antibiotics | | n.s. | Sig | n.s. | n.s. | n.s. | Sig | n.s | No | No | |
| Fluspirilene | Antiarrhythmic | | Sig | n.s. | n.s. | n.s. | n.s. | n.s. | Sig | Yes ^[1]^ | No | |
| Erlotinib | Antineoplastic | | Sis | Sig | n.s. | n.s. | n.s. | Sig | n.s. | No | No | |
| Encenicline | Nicotinic Agonists | | Sig | n.s. | n.s. | n.s. | n.s. | n.s. | Sig | No | No | |
| Deferasirox | Chelating Agents | | Sig | Sig | n.s. | n.s. | n.s. | n.s. | Sig | No | No | |
| Bumetanide | Diuretic | | Sig | n.s. | n.s. | n.s. | n.s. | n.s. | Sig | No | No | |
| Bezafibrate | Hypolipidemic | | Sig | n.s. | n.s. | n.s. | n.s. | n.s. | Sig | Yes ^[2]^ | No | |
| Apremilast | immunomodulatory | | n.s. | Sig | n.s. | n.s. | n.s. | n.s. | Sig | Yes ^[3]^ | No | |
| IHZ | Benzenoids | | Sig | n.s. | n.s. | Sig | n.s. | n.s. | n.s. | No | No | |

[1] Tabatabaei Dakhili SA, Greenwell AA, Yang K, et al. The Antipsychotic Dopamine 2 Receptor Antagonist Diphenylbutylpiperidines Improve Glycemia in Experimental Obesity by Inhibiting Succinyl-CoA:3-Ketoacid CoA Transferase. Diabetes. 2023 Jan 1;72(1):126-134.

[2] Franko A, Neschen S, Rozman J, et al. Bezafibrate ameliorates diabetes via reduced steatosis and improved hepatic insulin sensitivity in diabetic TallyHo mice. Mol Metab. 2017 Jan 6;6(3):256-266.

[3] Gomaa AA, Farghaly HSM, Ahmed AM, et al. Intermittent treatment with Apremilast, a phosphodiesterase-4 inhibitor, ameliorates Alzheimer's-like pathology and symptoms through multiple targeting actions in aged T2D rats. Int Immunopharmacol. 2023 Apr;117:109927.
